# Supplementary material for: Drug Resistance Patterns of Commonly Used Antibiotics for the Treatment of Helicobacter pylori Infection among South Asian Countries: A Systematic Review and Meta-Analysis
Source: Trop Med Infect Dis. 2023 Mar 14;8(3):172. doi: 10.3390/tropicalmed8030172 (PMC10051479; doi:10.3390/tropicalmed8030172)
Supplement: Supplementary file 1 [file tropicalmed-08-00172-s001.zip › tropicalmed-2217518-supplementary.pdf]

Supplementary Materials of Drug Resistance Patterns of Commonly Used Antibiotics for the Treatment of Helicobacter pylori Infection among South Asian Countries: A Systematic Review and Meta-Analysis

Supplementary Materials 1. PRISMA Checklist

Supplementary Materials 2. Search Strategy

Supplementary Materials 3. Quality Assessment (Newcastle-Ottawa scale)

Supplementary Materials 4. Countries of South Asia

Supplementary Materials 5. Subgroup Analysis

Supplementary Materials 6. Sensitivity Analysis

Supplementary Materials 7. Publication Bias

Supplementary Materials 1. PRISMA Checklist

Section and Topic	Item #	Checklist item	Location where item is reported
**TITLE**			
Title	1	Identify the report as a systematic review.	Page 1
**ABSTRACT**			
Abstract	2	See the PRISMA 2020 for Abstracts checklist.	Page 2
**INTRODUCTION**			
Rationale	3	Describe the rationale for the review in the context of existing knowledge.	Page 3
Objectives	4	Provide an explicit statement of the objective(s) or question(s) the review addresses.	Page 3
**METHODS**			
Eligibility criteria	5	Specify the inclusion and exclusion criteria for the review and how studies were grouped for the syntheses.	2.3
Information sources	6	Specify all databases, registers, websites, organisations, reference lists and other sources searched or consulted to identify studies. Specify the date when each source was last searched or consulted.	2.2
Search strategy	7	Present the full search strategies for all databases, registers and websites, including any filters and limits used.	2.2
Selection process	8	Specify the methods used to decide whether a study met the inclusion criteria of the review, including how many reviewers screened each record and each report retrieved, whether they worked independently, and if applicable, details of automation tools used in the process.	2.4

Section and Topic	Item #	Checklist item	Location where item is reported
Data collection process	9	Specify the methods used to collect data from reports, including how many reviewers collected data from each report, whether they worked independently, any processes for obtaining or confirming data from study investigators, and if applicable, details of automation tools used in the process.	2.4
Data items	10a	List and define all outcomes for which data were sought. Specify whether all results that were compatible with each outcome domain in each study were sought (e.g. for all measures, time points, analyses), and if not, the methods used to decide which results to collect.	2.6
	10b	List and define all other variables for which data were sought (e.g. participant and intervention characteristics, funding sources). Describe any assumptions made about any missing or unclear information.	2.6
Study risk of bias assessment	11	Specify the methods used to assess risk of bias in the included studies, including details of the tool(s) used, how many reviewers assessed each study and whether they worked independently, and if applicable, details of automation tools used in the process.	2.7
Effect measures	12	Specify for each outcome the effect measure(s) (e.g. risk ratio, mean difference) used in the synthesis or presentation of results.	2.8
Synthesis methods	13a	Describe the processes used to decide which studies were eligible for each synthesis (e.g. tabulating the study intervention characteristics and comparing against the planned groups for each synthesis (item #5)).	2.8
	13b	Describe any methods required to prepare the data for presentation or synthesis, such as handling of missing summary statistics, or data conversions.	-
	13c	Describe any methods used to tabulate or visually display results of individual studies and syntheses.	2.8
	13d	Describe any methods used to synthesize results and provide a rationale for the choice(s). If meta-analysis was performed, describe the model(s), method(s) to identify the presence and extent of statistical heterogeneity, and software package(s) used.	2.8
	13e	Describe any methods used to explore possible causes of heterogeneity among study results (e.g. subgroup analysis, meta-regression).	2.8
	13f	Describe any sensitivity analyses conducted to assess robustness of the synthesized results.	2.8
Reporting bias assessment	14	Describe any methods used to assess risk of bias due to missing results in a synthesis (arising from reporting biases).	-
Certainty assessment	15	Describe any methods used to assess certainty (or confidence) in the body of evidence for an outcome.	-
**RESULTS**			

Section and Topic	Item #	Checklist item	Location where item is reported
Study selection	16a	Describe the results of the search and selection process, from the number of records identified in the search to the number of studies included in the review, ideally using a flow diagram.	3.1
	16b	Cite studies that might appear to meet the inclusion criteria, but which were excluded, and explain why they were excluded.	3.1
Study characteristics	17	Cite each included study and present its characteristics.	3.1
Risk of bias in studies	18	Present assessments of risk of bias for each included study.	3.2
Results of individual studies	19	For all outcomes, present, for each study: (a) summary statistics for each group (where appropriate) and (b) an effect estimate and its precision (e.g. confidence/credible interval), ideally using structured tables or plots.	3.3
Results of syntheses	20a	For each synthesis, briefly summarise the characteristics and risk of bias among contributing studies.	3.2
	20b	Present results of all statistical syntheses conducted. If meta-analysis was done, present for each the summary estimate and its precision (e.g. confidence/credible interval) and measures of statistical heterogeneity. If comparing groups, describe the direction of the effect.	3.3
	20c	Present results of all investigations of possible causes of heterogeneity among study results.	3.4
	20d	Present results of all sensitivity analyses conducted to assess the robustness of the synthesized results.	3.5
Reporting biases	21	Present assessments of risk of bias due to missing results (arising from reporting biases) for each synthesis assessed.	-
Certainty of evidence	22	Present assessments of certainty (or confidence) in the body of evidence for each outcome assessed.	3.6
**DISCUSSION**			
Discussion	23a	Provide a general interpretation of the results in the context of other evidence.	4
	23b	Discuss any limitations of the evidence included in the review.	4
	23c	Discuss any limitations of the review processes used.	4
	23d	Discuss implications of the results for practice, policy, and future research.	4
**OTHER INFORMATION**			
Registration and protocol	24a	Provide registration information for the review, including register name and registration number, or state that the review was not registered.	reference number ID: CRD42021264656 PROSPERO

Section and Topic	Item #	Checklist item	Location where item is reported
	24b	Indicate where the review protocol can be accessed, or state that a protocol was not prepared.	PROSPERO
	24c	Describe and explain any amendments to information provided at registration or in the protocol.	-
Support	25	Describe sources of financial or non-financial support for the review, and the role of the funders or sponsors in the review.	No financial support
Competing interests	26	Declare any competing interests of review authors.	Not any
Availability of data, code and other materials	27	Report which of the following are publicly available and where they can be found: template data collection forms; data extracted from included studies; data used for all analyses; analytic code; any other materials used in the review.	Details available in the supplementary file

Supplementary Materials 2: Search Strategy

For Embase

#1: "Helicobacter pylori"
#2: "Helicobacter infection"
#3: “H. pylori”
#4: #1 OR #2 OR #3
#5: “antibiotic”
#6: “antibacterial”
#7: “antimicrobial”
#8: #5 OR #6 OR #7
#9: “resistance”
#10: #8 AND #9
#11: #4 AND #11
#12: (“Afghanistan” OR “Bangladesh” OR “Bhutan” OR “India” OR “Maldives” OR “Nepal” OR “Pakistan” OR “Sri Lanka”)
#13: #11 AND #12
Filters: human subjects

Supplementary Materials 3: Quality Assessment

NEWCASTLE - OTTAWA QUALITY ASSESSMENT SCALE (adapted for cross sectional studies)

Selection: (Maximum 4 stars)

1) Representativeness of the sample:

a) Truly representative of the average in the target population. * (all subjects or random sampling)
b) Somewhat representative of the average in the target population. * (nonrandom sampling)
c) Selected group of users.
d) No description of the sampling strategy.

2) Sample size:

a) Justified and satisfactory. *

b) Not justified.

3) Non-respondents:

a) Comparability between respondents and non-respondents' characteristics is established, and the response rate is satisfactory. *

b) The response rate is unsatisfactory, or the comparability between respondents and non-respondents is unsatisfactory.

c) No description of the response rate or the characteristics of the responders and the non-responders.

4) Ascertainment of the exposure (risk factor):

a) Validated measurement tool. **

b) Non-validated measurement tool, but the tool is available or described. *

c) No description of the measurement tool.

Comparability: (Maximum 2 stars)

1) The subjects in different outcome groups are comparable, based on the study design or analysis. Confounding factors are controlled.

a) The study controls for the most important factor (select one). *

b) The study control for any additional factor. *

Outcome: (Maximum 3 stars)

1) Assessment of the outcome:

a) Independent blind assessment. **

b) Record linkage. **

c) Self report. *

d) No description.

2) Statistical test:

a) The statistical test used to analyze the data is clearly described and appropriate, and the measurement of the association is presented, including confidence intervals and the probability level (p value). *

b) The statistical test is not appropriate, not described or incomplete.

Study	Selection (stars)		Comparability (stars)		Exposure (stars)		Total score (stars)		Mean score (stars)	Risk of bias	Include/Exclude
	Author (PP)	Author (SAM)	Author (PP)	Author (SAM)	Author (PP)	Author (SAM)	Author (PP)	Author (SAM)			
**Mujtaba et al.**	**3**	**3**	1	1	3	3	7	7	7	Low risk	Include
Khan et al.	3	3	1	1	2	2	6	6	6	Intermediate risk	Include
Gehlot et al.	3	3	1	1	2	2	6	6	6	Intermediate risk	Include
Aftab et al.	4	4	1	1	3	3	8	8	8	Low risk	Include
Pandey et al.	3	3	1	1	2	2	6	6	6	Intermediate risk	Include
Miftahussurer et al.	4	3	1	1	3	3	8	8	8	Low risk	Include
Mahant et al.	4	3	1	1	3	3	8	7	7.5	Low risk	Include
Malhotra et al.	3	4	1	1	3	3	7	8	7.5	Low risk	Include
Anis et al.	3	4	1	1	3	3	7	8	7.5	Low risk	Include
Datta et al.	3	3	1	1	3	3	7	7	7	Low risk	Include
Nahar et al.	4	3	1	1	3	3	8	7	7.5	High risk	Include
Hallur et al.	3	3	1	1	3	3	7	7	7	High risk	Include
Rajper et al.	3	3	1	1	3	3	7	7	7	Low risk	Include
Rasheed et al.	4	3	1	1	3	3	8	7	7.5	Low risk	Include
Shetty et al.	3	4	1	1	3	3	7	8	7.5	Low risk	Include
Singh et al.	3	3	1	1	3	3	7	7	7	Low risk	Include
Siddiqui et al.	4	4	1	1	3	3	8	8	8	Low risk	Include
Thyagarajan et al.	3	3	1	1	3	3	7	7	7	Low risk	Include
Vagarali et al.	3	3	1	1	3	3	7	7	7	Low risk	Include

Vilaichone et al.	4	3	1	1	3	3	8	7	7.5	Low risk	Include
Vilaichone et al.	4	3	1	1	3	3	8	7	7.5	Low risk	Include
Wani et al.	3	4	1	1	3	3	7	8	7.5	Low risk	Include
Yakoob et al.	3	3	1	1	2	3	6	7	6.5	Intermediate risk	Include

Supplementary Materials 4: Countries of South Asia

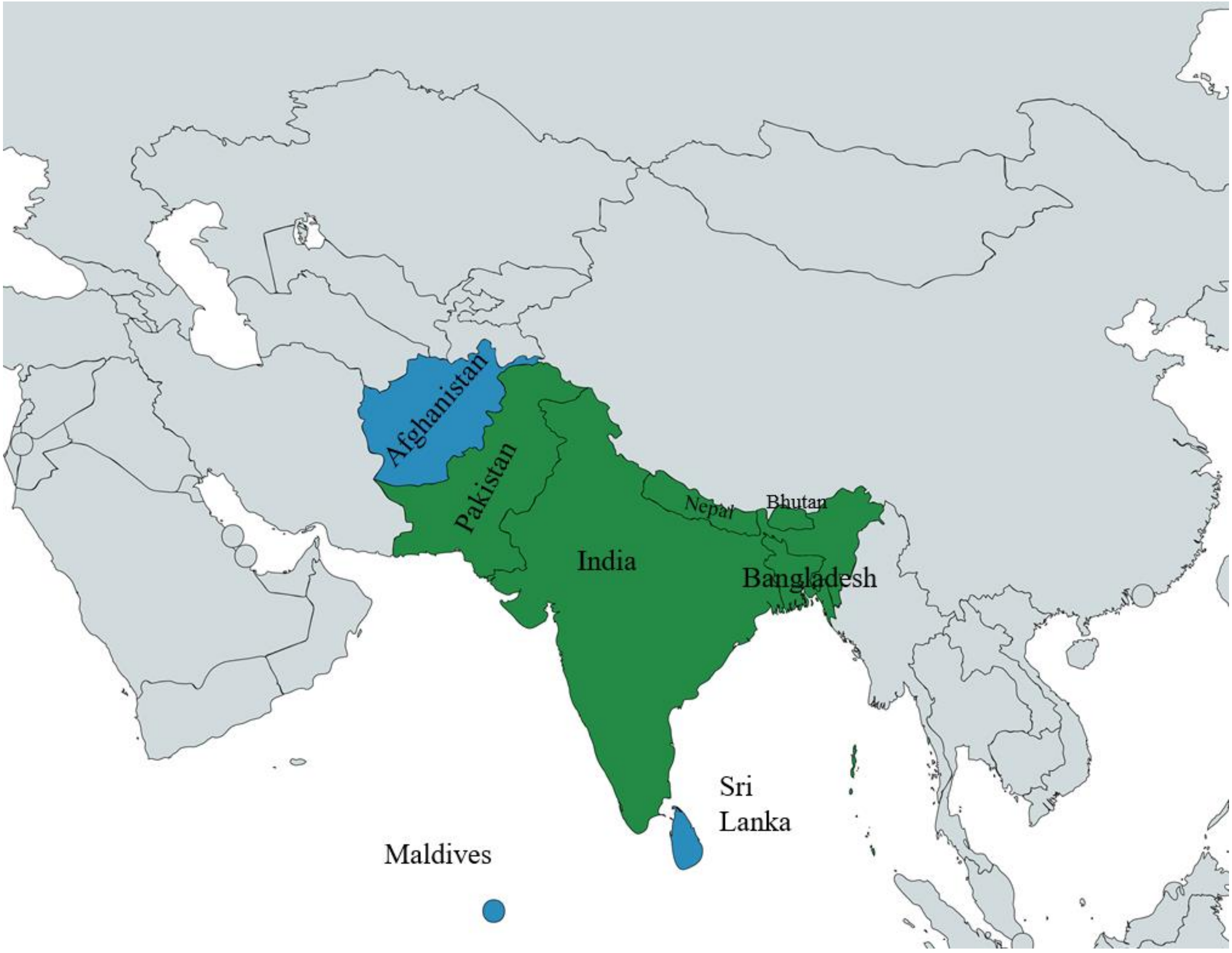

Figure. Countries of the South Asia.

(■) = Countries included in the meta-analysis.

(■) = Countries not included in the meta-analysis.

Supplementary Materials 5: Subgroup Analysis

Method of detection of H pylori and resistivity

	**Metronidazole**	**P value**	**Clarithromycin**	**P value**	**Tetracycline**	**P value**	**Amoxicillin**	**P value**	**Levofloxacin**	**P value**	**Ciprofloxacin**	**P value**
						**H pylori detection**						
**Combine**	0.874(0.82-0.92)	<0.001	0.38(0.01-0.75)	<0.001	0.187(0.001-0.37)	<0.001	0.36(0.03-0.76)	<0.001	0.34(0.05-0.61)	<0.001	0.02(0.007-0.04)	<0.001
**Culture**	0.57 (0.37-0.77)	<0.001	0.11(0.07-0.15)	<0.001	0.1(0.05-0.14)	<0.001	0.11(0.07-0.16)	<0.001	0.31(0.122-0.503)	<0.001	0.16(0.01-0.3)	<0.001
**H&E**	0.473(0.19-1.14)	<0.001	0.29(0.12-0.45)	0.002	0.05(0.01-0.1)	<0.001	0.08(0.03-0.12)	0.465	0.54(0.45-0.64)	<0.001	NA	

**H pylori resistance detection**												
**Combined**	0.89(0.677-1.105)	<0.001	0.18(0.07-0.44)	<0.001	0.01(0.00-0.02)	0.13	0.47(0.14-0.8)	<0.001	0.89(0.8-0.98)	<0.001	0.02(0.007-0.04)	<0.001
**Agar Dilution**	0.57(0.33-0.82)	<0.001	0.2(0.07-0.33)	<0.001	0.1(0.02-0.22)	0.01	0.058(0.001-0.11)	<0.001	0.21(0.002-0.43)	<0.001	NA	<0.001
**E test**	0.63(0.29-0.97)	<0.001	0.04(0.01-0.07)	<0.001	0.003(0.002-0.007)	<0.001	0.00(0.00-0.00)	0.39	0.19(0.06-0.33)	<0.001	0.063(0.02-0.15)	0.72
**PCR**	0.62(0.34-0.9)	<0.001	0.32(0.23-0.41)	0.002	0.051(0.01-0.11)	0.003	0.16(0.00-0.34)	<0.001	0.54(0.45-0.33)	<0.001	NA	<0.001
**Disk Diffusion**	0.85(0.69-1.0)	<0.001	0.56(0.02-1.11)	<0.001	0.44(0.17-1.06)	0.395	0.64(0.3-0.99)	<0.001	0.367(0.22-0.51)	0.27	0.26(0.00-0.72)	<0.001

Supplementary Materials 6: Sensitivity Analysis

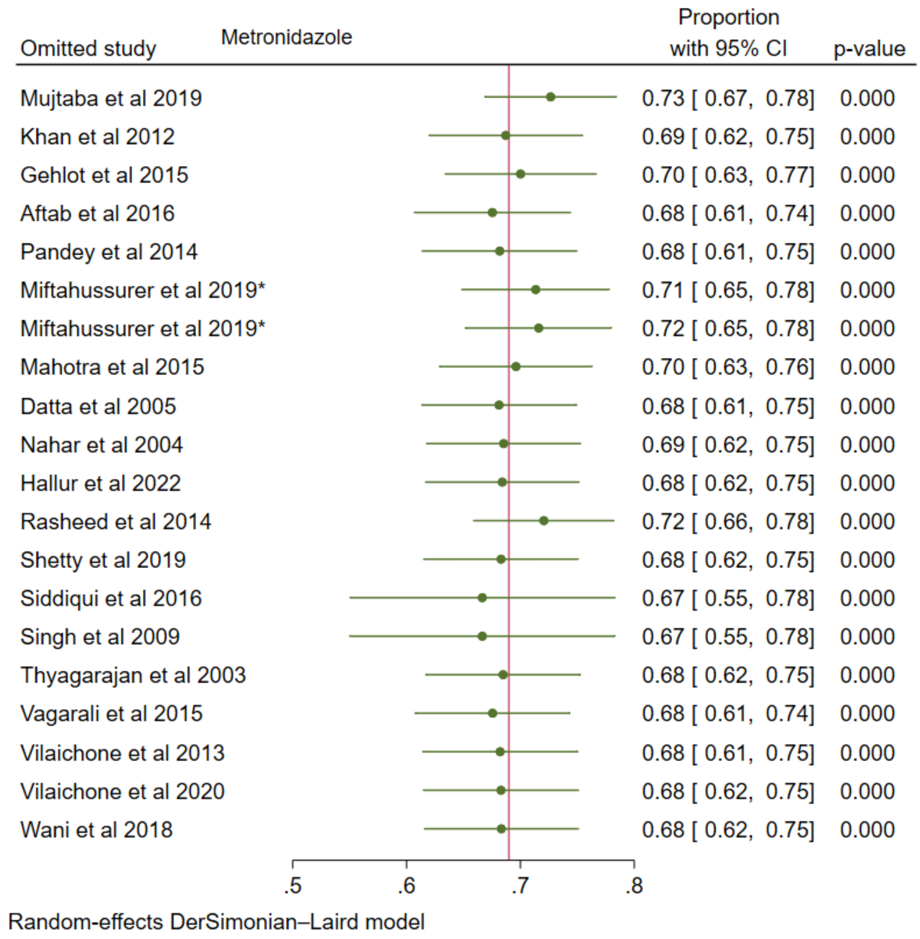

Figure S1. Sensitivity analysis for metronidazole.

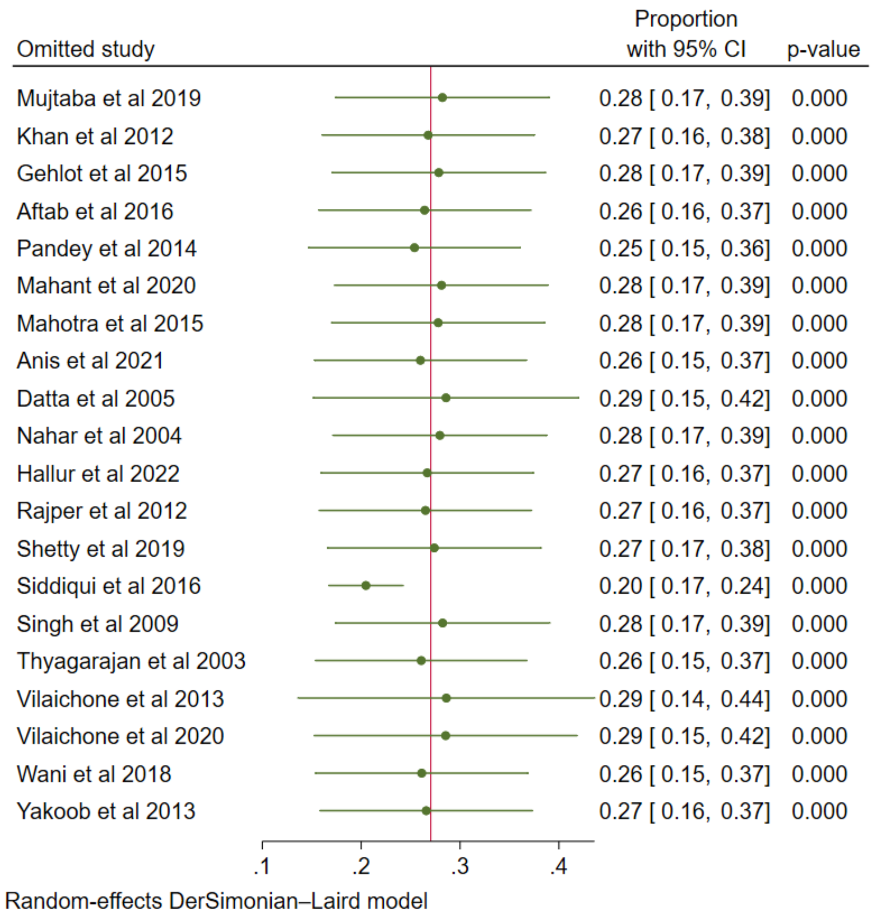

Figure S2. Sensitivity analysis for clarithromycin.

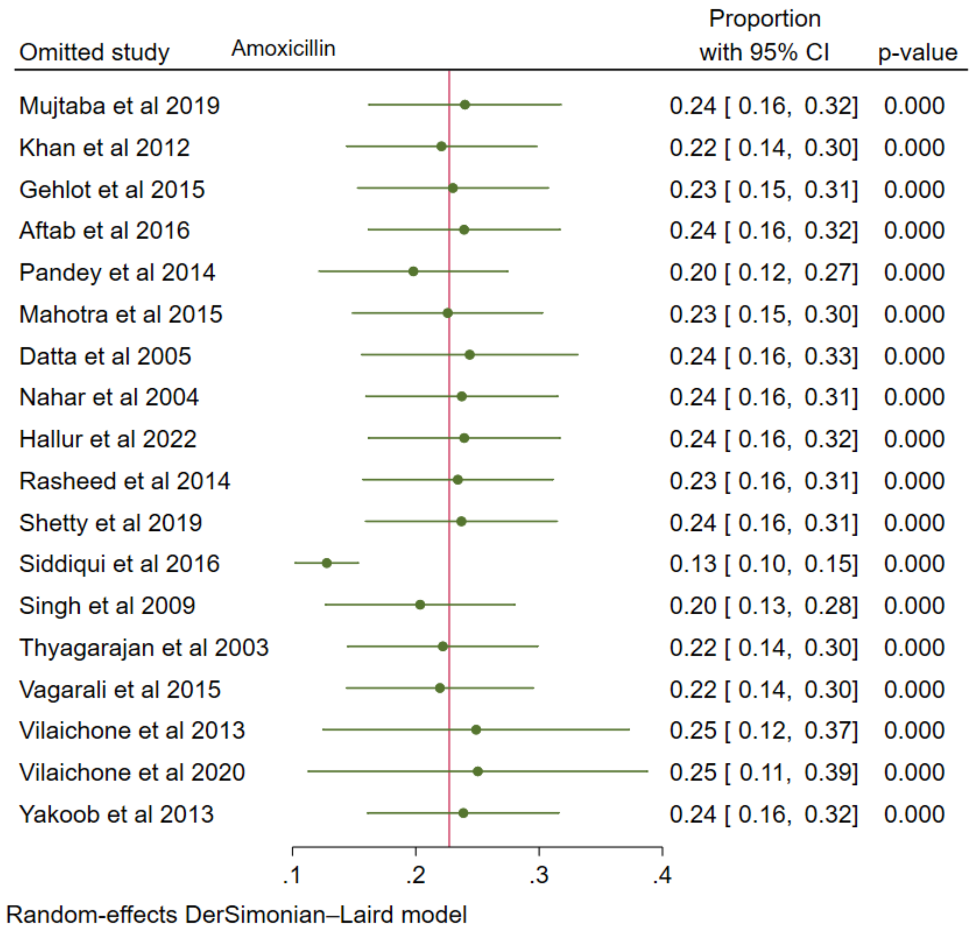

Figure S3. Sensitivity analysis for amoxicillin.

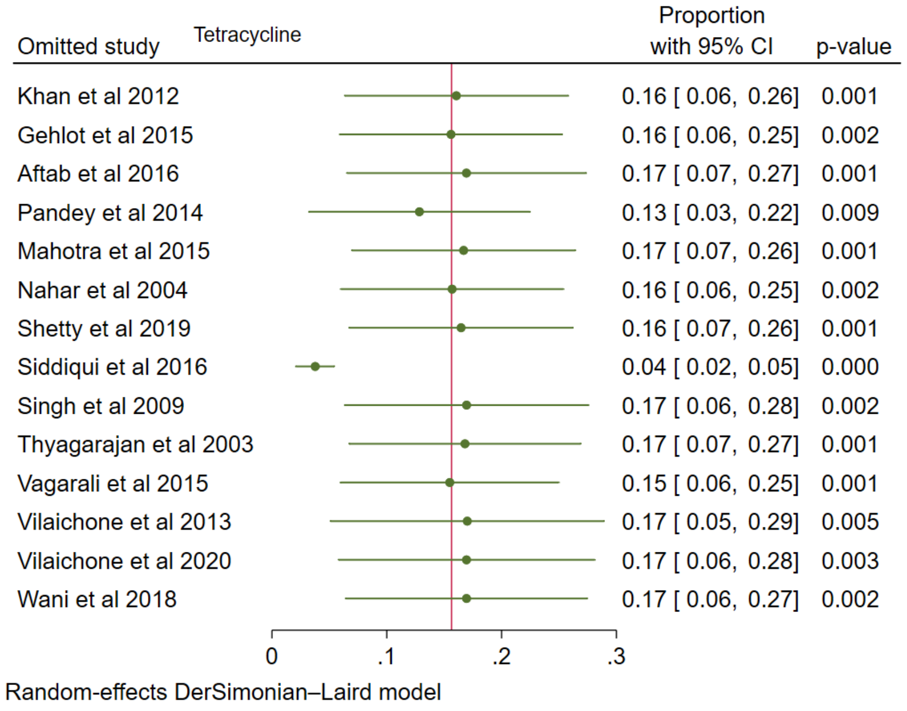

Figure S4. Sensitivity analysis for tetracycline.

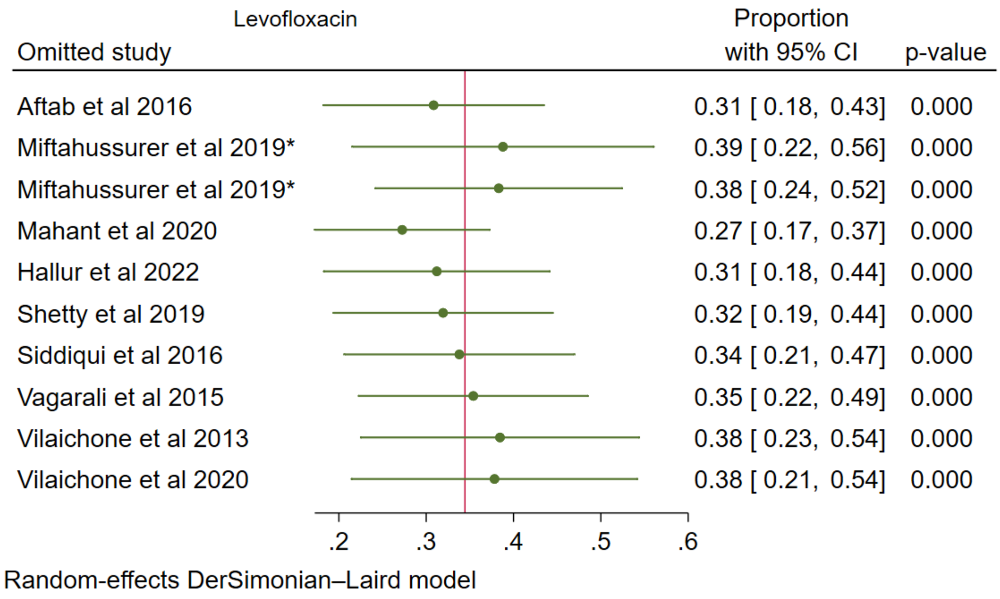

Figure S5. Sensitivity analysis for levofloxacin.

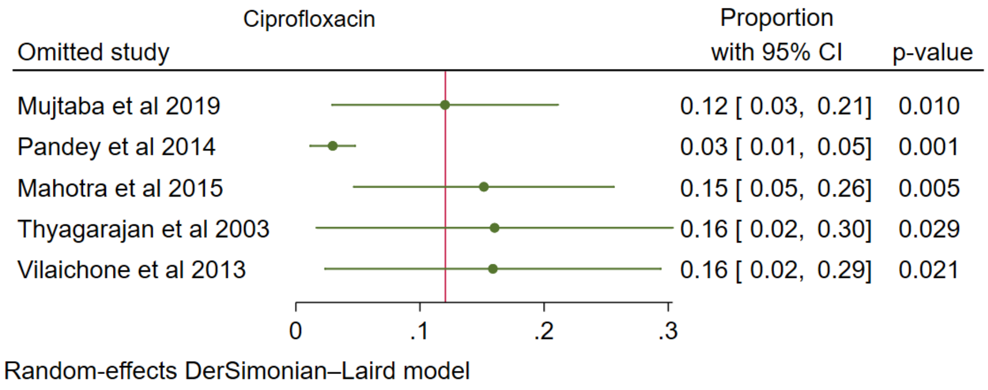

Figure S6. Sensitivity analysis for ciprofloxacin.

Supplmentary Mateirals 7: Publication Bias

A. Funnel Plots:

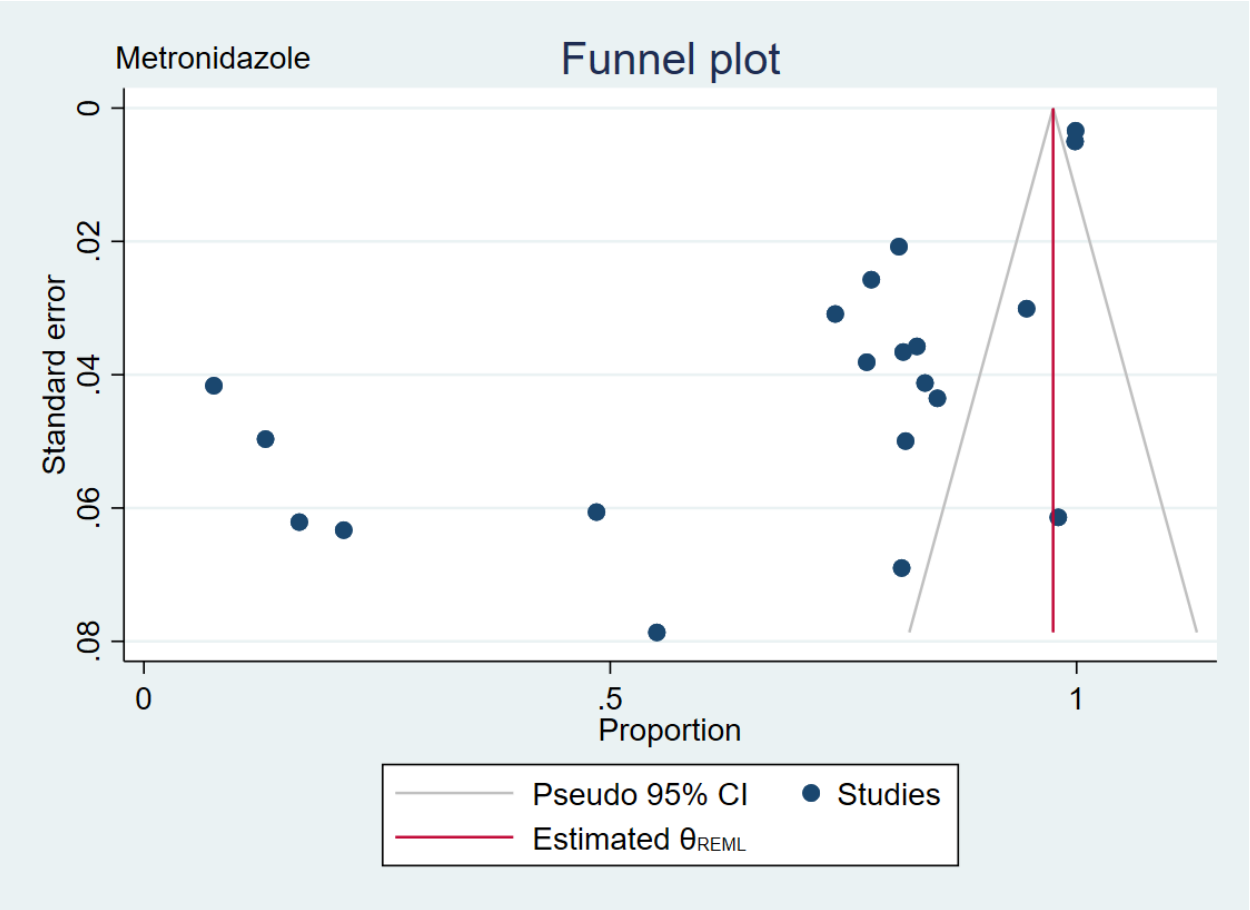

Figure S7. Funnel plot for metronidazole.

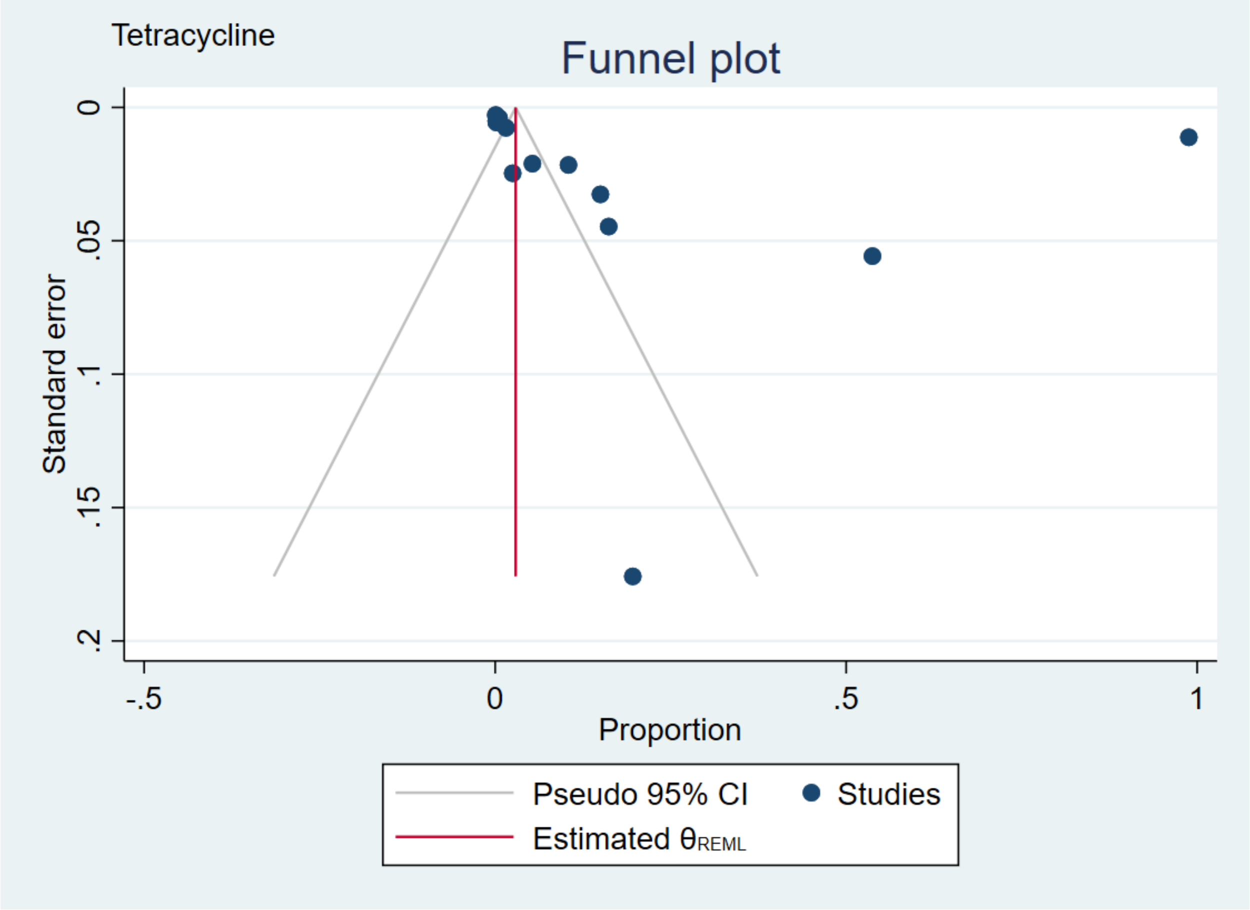

Figure S8. Funnel plot for tetracycline.

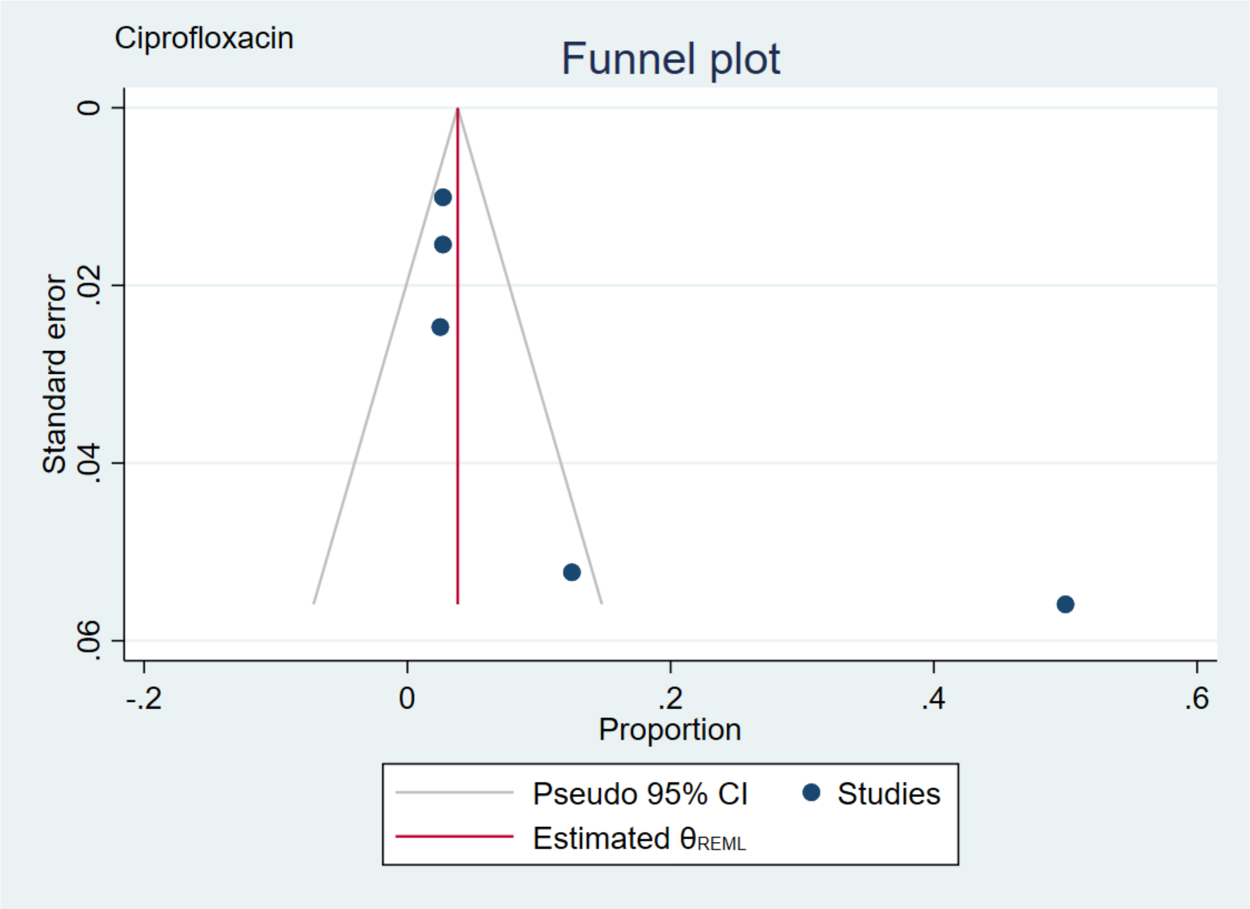

Figure S9. Funnel plot for ciprofloxacin.

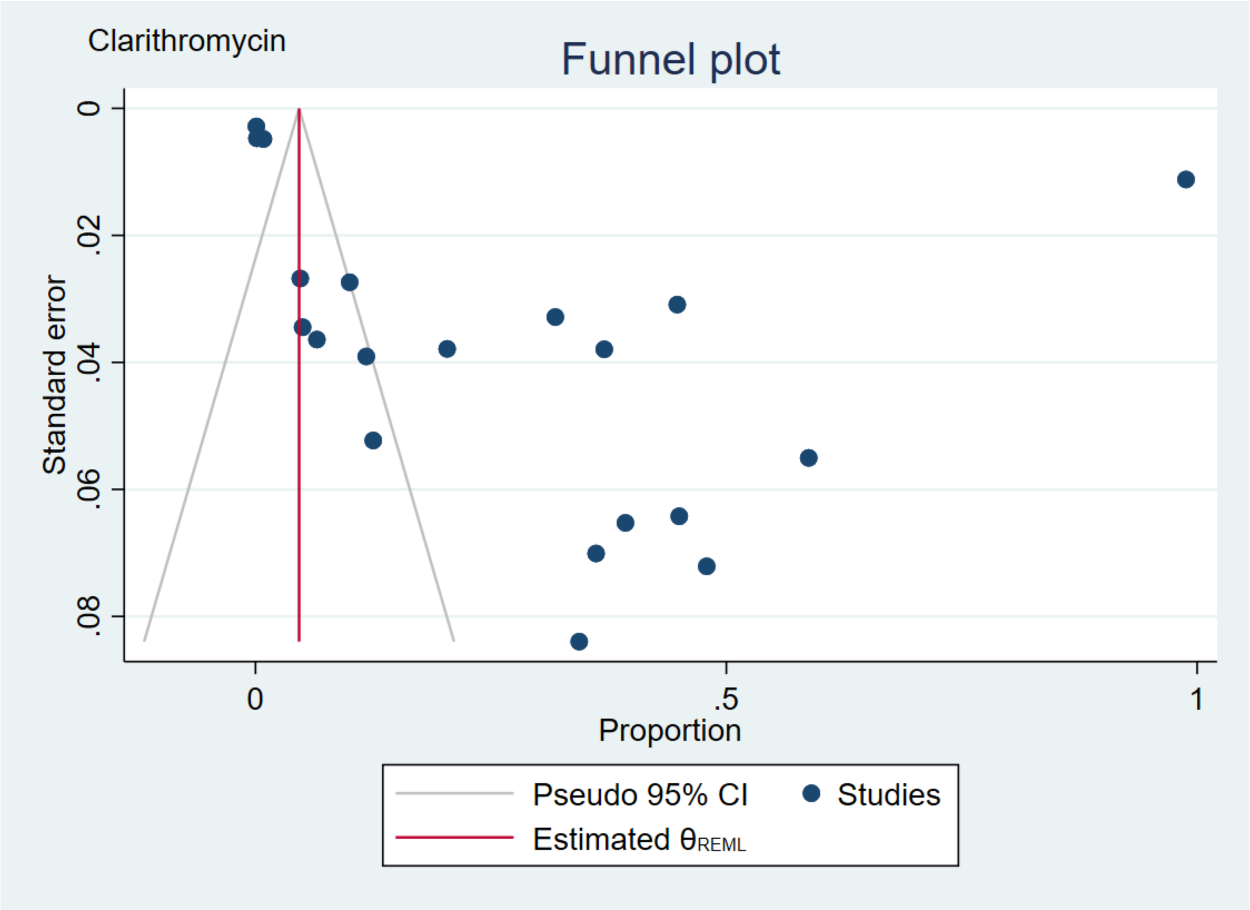

Figure S10. Funnel plot for clarithromycin.

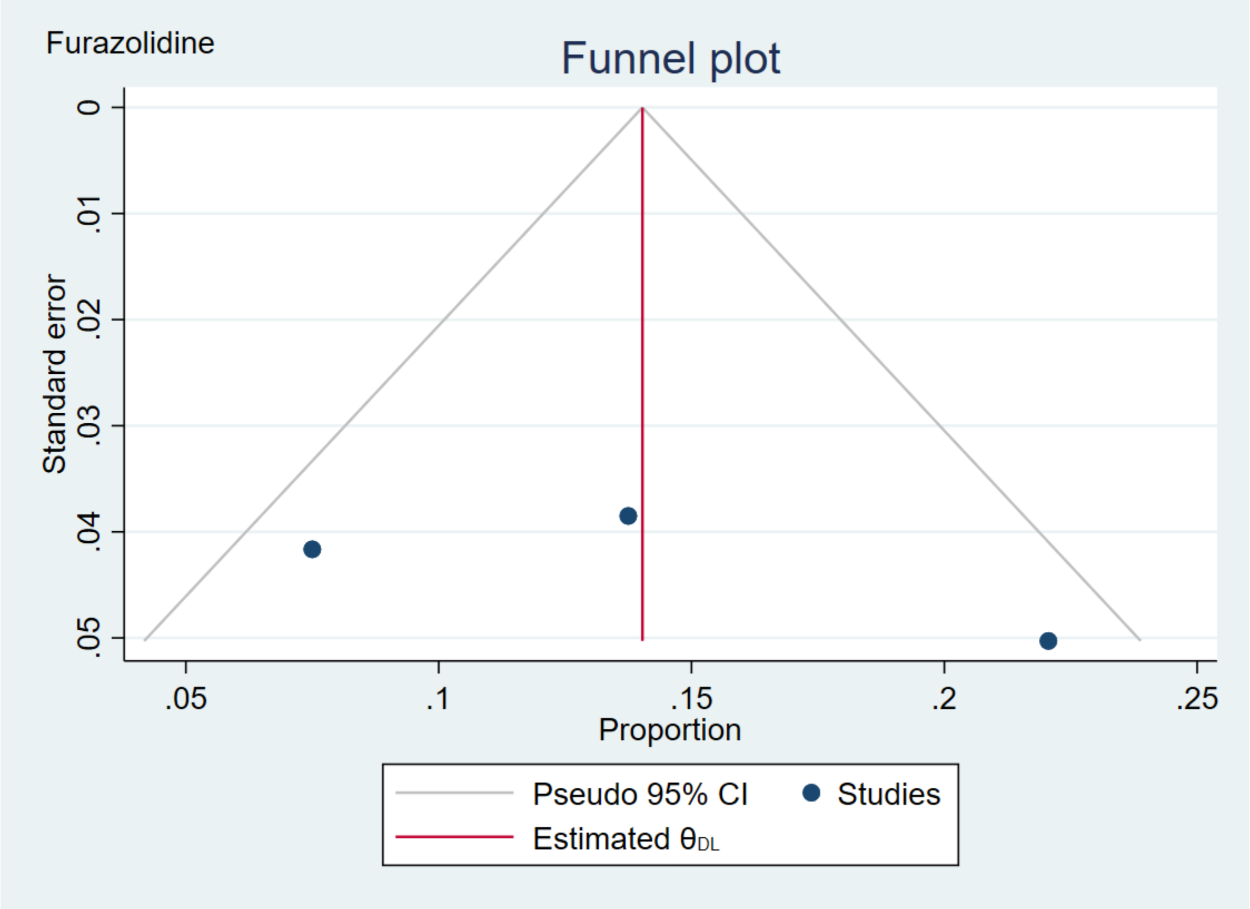

Figure S11. Funnel plot for furazolidone.

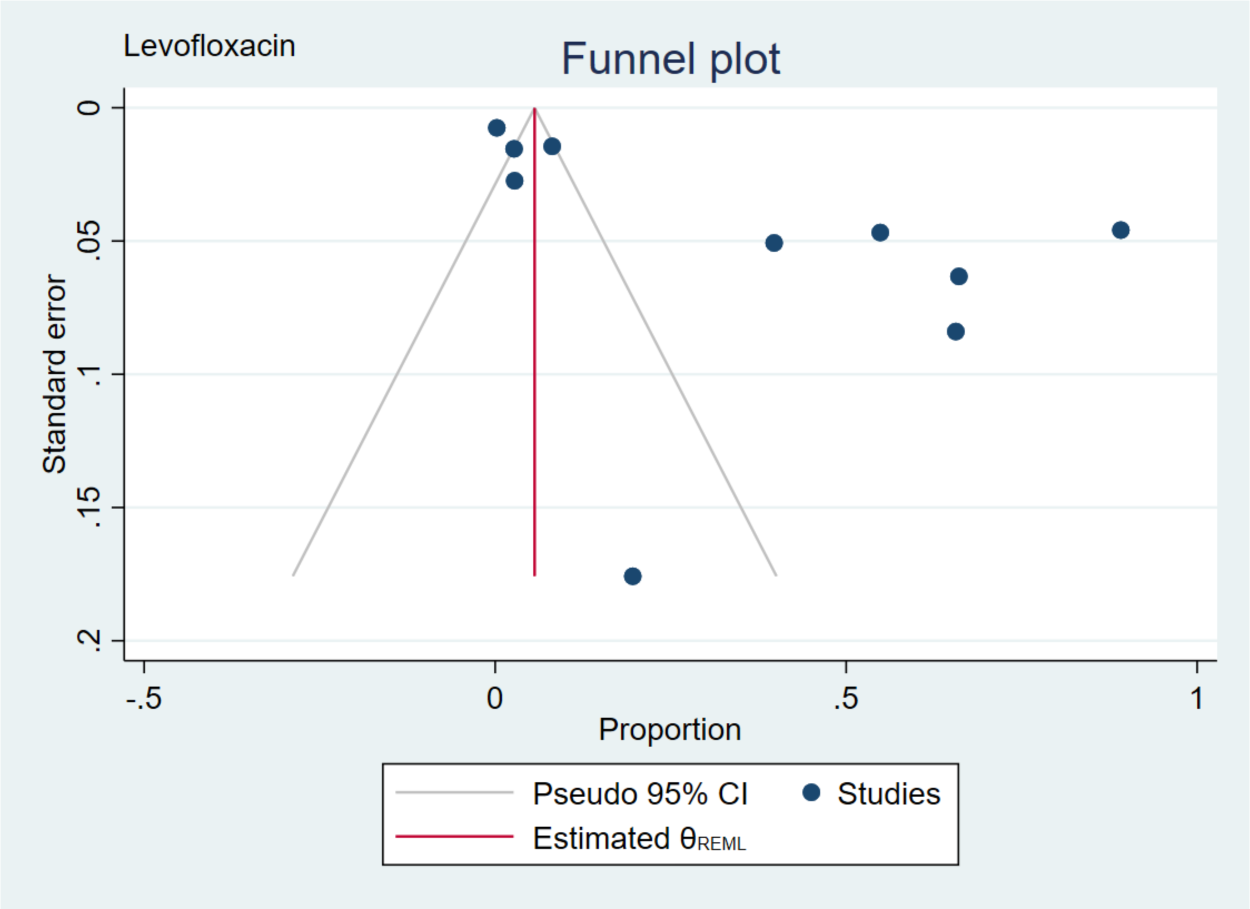

Figure S12. Funnel plot for levofloxacin.

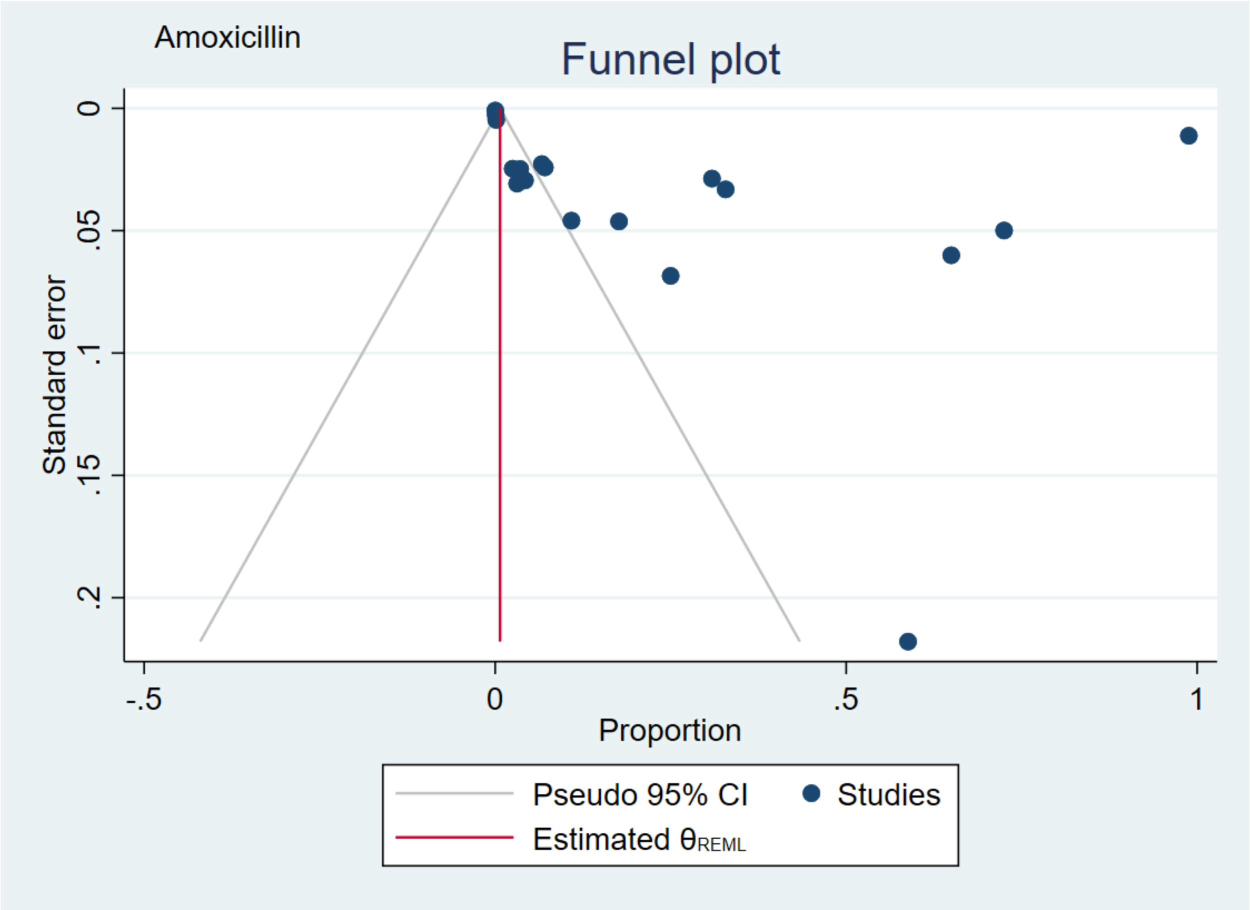

Figure S13. Funnel plot for amoxicillin.

B. Regression-based Egger test for small-study effects

Random-effects model

Method: DerSimonian–Laird

1. Metronidazole

H0: beta1 = 0; no small-study effects

beta1 = −7.60

SE of beta1 = 1.154

z = −6.59

Prob > |z| = 0.0000

2. Clarithromycin

H0: beta1 = 0; no small-study effects

beta1 = 3.76

SE of beta1 = 2.190

z = 1.72

Prob > |z| = 0.0860

3. Tetracycline

H0: beta1 = 0; no small-study effects

beta1 = 1.55

SE of beta1 = 1.975

z = 0.78

Prob > |z| = 0.4331

4. Amoxicillin

H0: beta1 = 0; no small-study effects

beta1 =	2.68
SE of beta1 =	1.778
z =	1.51
Prob > |z| =	0.1314

5. Ciprofloxacin

H0: beta1 = 0; no small-study effects

beta1 =	8.99
SE of beta1 =	2.946
z =	3.05
Prob > |z| =	0.0023

6. Levofloxacin

H0: beta1= 0; no small-study effects

beta1 =	2.28
SE of beta1 =	2.280
z =	1.00
Prob > |z| =	0.3182
